# Histopathologic Differences between Jewish and Arab Population in Israel at First-Time Presentation with Bladder Cancer

**DOI:** 10.1155/2017/8239601

**Published:** 2017-07-24

**Authors:** Ofir Avitan, Zaher Bahouth, Sarel Halachmi, Sagi Shprits, Ismail Masarwa, Edmond Sabo, Boaz Moskovitz, Ofer Nativ

**Affiliations:** ^1^Department of Urology, Bnai-Zion Medical Center, Haifa, Israel; ^2^Department of Pathology, Rambam Medical Center, Haifa, Israel

## Abstract

**Background:**

Pathology of urothelial carcinoma may vary in different populations at diagnosis. Our aim was to evaluate the histopathologic differences between Jewish and Arab patients in Israel at first diagnosis of urothelial cancer.

**Patients and Methods:**

We retrospectively collected data of all patients with confirmed urothelial cancer, treated at our department between January 2010 and January 2015. We examined the distribution of the histopathologic data among the studied populations. To compare the categorical variables we used the Chi-Square Pearson test. Comparison of independent variables was made by Student's *t*-test. *P* value below 0.05 was considered significant.

**Results:**

The study group included 413 patients, 345 Jews and 68 Arabs. The major differences were that Arab patients were younger (62.61 versus 68.55 years, *P* = 0.001), had more aggressive tumors that were detected at a more advanced stage, and had also a higher rate of metastatic disease (7.4% versus 3.2%, *P* = 0.05). Nonurothelial cell tumors were 2.3 times more prevalent in Arab population. Unlike Jewish population, Arab women had higher rate of invasive/metastatic disease compared with Arab men (40% versus 22.4%).

**Conclusion:**

At time of diagnosis the tumors were more aggressive in Arab patients, especially in Arab women. The reasons for those differences constitute a target for a separate research. These results should have an impact on prevention medicine and education of physicians treating mixed populations.

## 1. Introduction

In Israel approximately 1100 new cases of bladder cancer are diagnosed each year [1]. There are studies in the medical literature describing different pathological presentation in different populations [2–4]; however differences between Jews and Arabs were never studied. The aim of our study was to compare clinical and histopathology characteristics in patients with urothelial cancer at first presentation between Arab and Jewish patients diagnosed and treated at our medical center.

## 2. Patients and Methods

The study was approved by the Ethics Committee of the Bnai-Zion Medical Center. It is a retrospective, single center study where all information was obtained from the medical records of the studied patients.

### 2.1. Patients

The study population included 413 patients who presented with bladder cancer between January 2010 and January 2015 to the urology department at Bnai-Zion Medical Center, Haifa, Israel. All patients had pathologically confirmed urothelial cell carcinoma of the urinary bladder based on transurethral resection specimen. Patients with bladder malignancy originated from outside the urinary system or unequivocal diagnosis were excluded. To determine extent of disease patients underwent either CT Urography or MR Urography. The data was collected from medical records and included gender, origin (Jewish or Arab), age at first presentation, tumor type, grade, and stage.

### 2.2. Statistical Analysis

Processing and analyzing the data were done using SPSS version 20 software (IBM, CA, USA). We examine the distribution of data using Kolmogorov-Smirnov test. To compare the categorical variables we used the Chi-Square Pearson test. Independent variables were by using the Student *t*-test. The continuous variables were described by mean and standard deviation. The noncontinuous variables were described as percentages. The result of *P* value below 0.05 was considered significant.

## 3. Results

The study group included 413 patients, of which 68 were of Arab origin and 345 of Jewish origin; there were 75 women and 338 men, with mean age of 67.59 ± 12.97 years.

Table 1 presents the pathological and demographic data of all patients according to their origin.

Patients of Arabic origin presented at a younger age compared to Jewish patients (62.6 years versus 68.5 years, *P* = 0.001). No significant difference was noted between the studied populations in male to female ratio.

At diagnosis Arab patients had a more aggressive disease as evident by a slightly higher grade (60.3% versus 50.1%, *P* = 0.14), more advanced stage (25% versus 13.3% of muscle invasive disease, *P* = 0.025), and 2.3-fold increase in the rate of metastatic disease (7.4% versus 3.2%, *P* = 0.025). Although the rate of Arab patients diagnosed with nonpure urothelial carcinoma was higher than that detected in Jewish cases (7.4% versus 3.2%), these differences did not reach statistical significance, *P* = 0.082.

When the association of tumor characteristics and gender was studied in the two populations (Table 2), no difference between men and women of Jewish origin was noted. However, a fourfold increase of nonpure urothelial tumors was observed in Arab women. It was also found that at time of diagnosis 40% of Arab women had muscle invasive tumor compared with 22.4% among Arab men. These rates are higher than those found among Jewish men and women (14% and 13.2%, resp.).

## 4. Discussion

Our study demonstrated significant differences between two populations living in the same country regarding the presentation of urothelial cancer at diagnosis. Furthermore significant intrapopulation differences were noted between female and male Arabs. To our knowledge this is the first study that examined the characteristics of urothelial carcinoma in Jewish and Arab populations at diagnosis, in Israel.

The most prominent findings in our study are as follows:

1. Arab patients are diagnosed at younger ages (mean 62.61 years).
2. Bladder cancer in the Arab population is of higher grade than in Jews.
3. Patients of Arab origin were diagnosed at a higher stage including systemic dissemination.
4. The rate of nonpure urothelial cell carcinoma was higher in the Arab population, especially among women.

Different biological behavior in different races living in the same country was reported by Prout Jr. et al. [5] showing early age appearance in African Americans in the US. The percentage of African Americans patients under the age of 50 years at diagnosis was 53.3%, compared with 44.7% in white patients.

Concerning higher tumor grade at diagnosis in the Arab population, similar difference in tumor biology was found among African American patients compared with white patients [6, 7].

Arab patients were diagnosed at a higher stage including systemic dissemination. The difference in the proportion of muscle invasive disease was more prominent in Arab women compared to Arab men and Israeli women. Ethnic differences with respect to stage distribution at diagnosis were also reported by Yee et al. [8]; they summarized data of 185,002 patients showing twofold increase of metastatic disease in African American compared with white patients. These differences have an impact on patients' survival. Based on data obtained from Netherlands Cancer Registry, Mungan et al. [9] have shown that Dutch female has a higher rate of muscle invasive urothelial cell carcinoma compared with male patients.

The rate of nonpure urothelial cell carcinoma was higher in the Arab population, especially among women. Racial differences in the distribution of bladder cancer types were also described by Prout Jr. et al. [5] that reported a higher rate of bladder cancer histological variants among blacks compared with whites (17.9% versus 3.3%, resp.). A typical example of differences in the distribution of histological subgroups of urothelial tumors can be found in Egypt, where the rate of lesions with abnormal histology, particularly squamous urothelial carcinoma, is described in up to 73% of all diagnosed cases [10].

Possible explanations for the differences mentioned include influences of nutrition, smoking, residential areas, genetics, and a tendency to use alternative therapies. One cannot exclude also cultural causes that may have contributed to a delayed diagnosis especially in women.

Additional causes for the differences in histopathologic features between Jewish and Arab patients may be related to lower socioeconomic status that in general was more frequent in the latter group.

One may speculate that delayed diagnosis may also contribute to the worse histopathologic feature in Arab women found in our study.

There are strict differences in smoking habits between Jews and Arabs in Israel [11]. The latest report of the Israeli health service demonstrated higher smoking rate in the Arab population, 25.2% versus 18.5% in Jews. Within Arabs, smoking rate in men was 43.9% and 6.7% in women.

The above data may explain the higher rate of urothelial cancer in Arab males; the higher rate of cancer in Arab women could also be attributed to passive smoking; however other factors needed to be investigated to understand this biological behavior.

The study has several limitations including relatively small study group, particularly of the Arab population. Data was collected from a single center and may not represent the whole population of the state of Israel. There is no long-term clinical follow-up that could shed light on the clinical significance of the histological differences at presentation.

## 5. Conclusions

The study demonstrated histopathological differences between Jewish and Arab patients at first presentation of urothelial carcinoma. Arab patients have higher grade tumors, more advanced stage disease, and increased rate of nonpure transitional cell variants. These differences are more evident among Arab women.

## Figures and Tables

**Table 1:** Comparison of Jewish and Arabs: tumor differentiation, invasiveness, and aggressiveness.

	Arabs (*n* = 68)	Jewish (*n* = 345)	Entire study group (*n* = 413)	*P* value	OR
*Age [years]*	62.68 ± 12.71	68.55 ± 12.82	67.59 ± 12.97	0.001	
*Gender*					
Men (*n* = 338)	58 (85.3%)	280 (81.2%)	338 (81.8%)	0.33	
Women (*n* = 75)	10 (14.7%)	65 (18.8%)	75 (18.2%)		
Men : women ratio	5.8 : 1	4.3 : 1	4.5 : 1		
*Tumor type*					
Transitional urothelial	63 (92.6%)	334 (96.8%)	397 (96.1%)	0.082	3.3
Other	5 (7.4%)	11 (3.2%)	16 (3.9%)		
*Malignancy grade*					
Low grade	27 (39.7%)	172 (49.9%)	199 (48.2%)	0.14	1.5
High grade	41 (60.3%)	173 (50.1%)	214 (51.8%)		
*Stage*					
Noninvasive	51 (75%)	299 (86.7%)	350 (84.7%)	0.025	2.2
Invasive	17 (25%)	46 (13.3%)	63 (15.3%)		
Metastatic	5 (7.4%)	11 (3.2%)	16 (3.9%)		

**Table 2:** Comparison of Jewish and Arabs/Men and Women: tumor differentiation, invasiveness, and aggressiveness.

	Men (*n* = 338)		OR	*P*	Women (*n* = 75)		*P*	OR
	Jewish	Arab			Jewish	Arab		
*Tumor type*								
Transitional urothelial	274 (98%)	55 (95%)	2.49	0.188	61 (94%)	8 (80%)	0.18	3.4
Other	6 (2%)	3 (5%)			4 (6%)	2 (20%)		
*Malignancy grade*								
Low grade	139 (49.6%)	23 (40%)	1.5	0.194	33 (50.7%)	4 (40%)	0.74	1.54
High grade	141 (50.4%)	35 (60%)			32 (49.3%)	6 (60%)		
*Stage*								
Noninvasive	243 (86.8%)	45 (77.6%)	1.9	0.1	56 (86%)	6 (60%)	0.06	4.14
Invasive	37 (13.2%)	13 (22.4%)			9 (14%)	4 (40%)		
